# Correction to “Safety, tolerability, pharmacokinetic/pharmacodynamic characteristics of bersiporocin, a novel prolyl‐tRNA synthetase inhibitor, in healthy subjects”

**DOI:** 10.1111/cts.13783

**Published:** 2024-04-01

Park MY, Bae S, Heo JA, et al. Safety, tolerability, pharmacokinetic/pharmacodynamic characteristics of bersiporocin, a novel prolyl‐tRNA synthetase inhibitor, in healthy subjects. *Clin. Transl. Sci*. 2023;16(7):1163–1176. doi: 10.1111/cts.13518. Epub 2023 Apr 24. PMID: 37095713; PMCID: PMC10339703.

The authors are adding the following information to the title page:

“Min Young Park and Sungyeun Bae contributed equally to this work.”

These authors made significant joint contributions to the work and should be acknowledged equally.

